# Supplementary material for: Phase-referenced nonlinear spectroscopy of the α-quartz/water interface
Source: Nat Commun. 2016 Dec 13;7:13587. doi: 10.1038/ncomms13587 (PMC5159844; doi:10.1038/ncomms13587)
Supplement: Supplementary Information — Supplementary Figures 1-8, Supplementary Table 1, Supplementary Note 1 and Supplementary References [file ncomms13587-s1.pdf]

Supplementary Information.

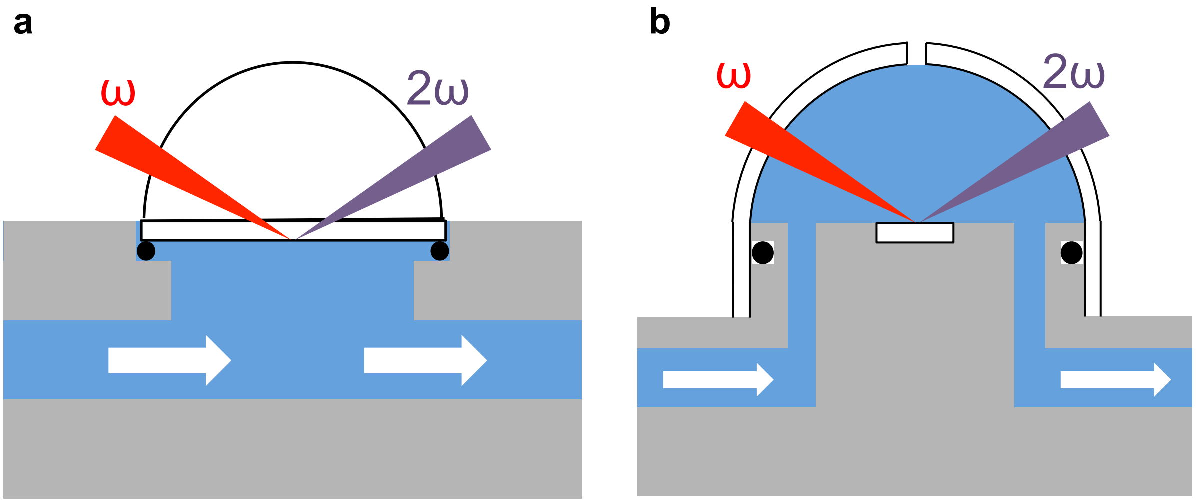

Supplementary Figure 1 | (a) Schematic depictions of the total-internal or near total-internal reflection geometry. (**b**) Schematic depictions of the external reflection geometry. Internal reflection has been used generally by our lab[1,2] and others[3] as it produces a higher signal intensity due to higher Fresnel coefficients.[4] We utilize fused silica hemispheres in order to ensure high transmission coefficients and retain the input polarization at any input angle. To study surfaces besides fused silica in this geometry, disks of material such as muscovite[5] can be clamped between the hemisphere and the aqueous phase. However, the non-centrosymmetric nature of α-quartz produced an overwhelmingly large bulk signal in this geometry, motivating the switch to the external reflection geometry (see Table S1).

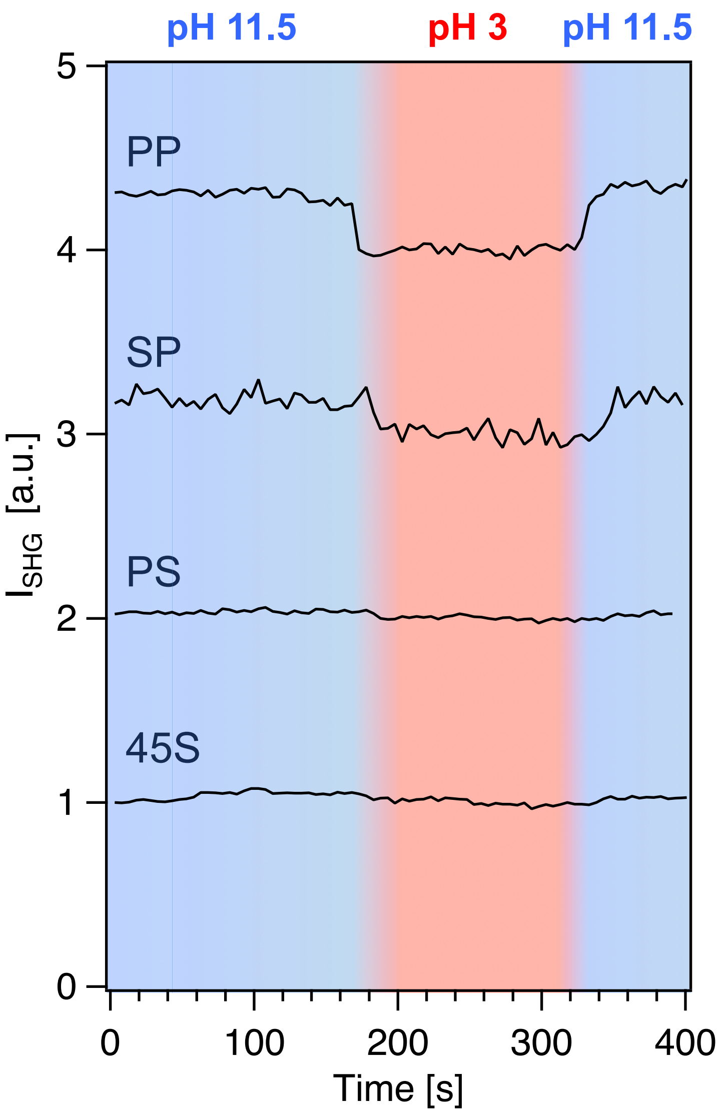

Supplementary Figure 2 | Polarization combination dependence of the α-quartz I_{SHG} response to pH jump experiments (the first index represents input polarization, the second represents output polarization; 45 represents mixed polarization). The crystal was oriented at 30° and the traces are normalized to their intensities at low pH; each is offset by 1 unit. The PP polarization combination, showing the highest sensitivity to interfacial potential, was used in all other experimental results reported unless otherwise specified.

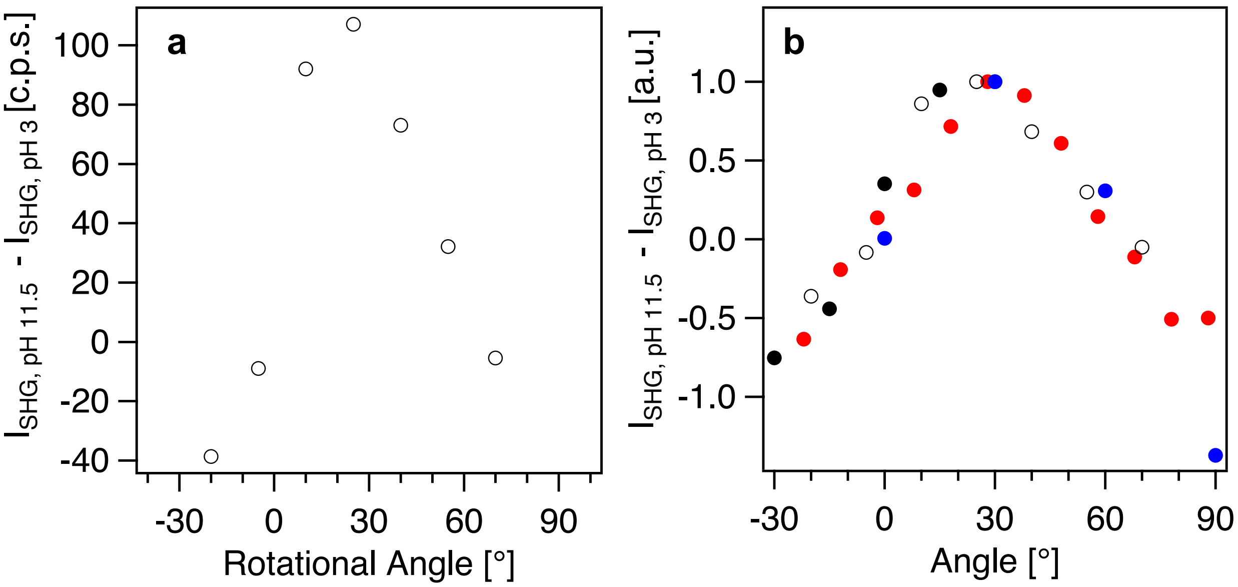

Supplementary Figure 3 | (a) The difference in I_{SHG} at low and high pH conditions, ΔI_{SHG}, as a function of rotational angle of the α-quartz crystal for the data set included in Figure 3 in the main text. (**b**) ΔI_{SHG}, as a function of rotational angle of the α-quartz crystal for all experiments carried out on three different samples from different suppliers, normalized to the maximum difference obtained in each run (blue circles, PM Optics; open black circles, Meller Optics Experimental Run 1; closed black circles, Meller Optics Experimental Run 2; red circles, Knight Optical). The same overall pattern holds accross of the samples of little to no change at 0° and 60°, a maximum of constructive interference at 30°, and a maximum of destructive interference at -30° (= 90°).

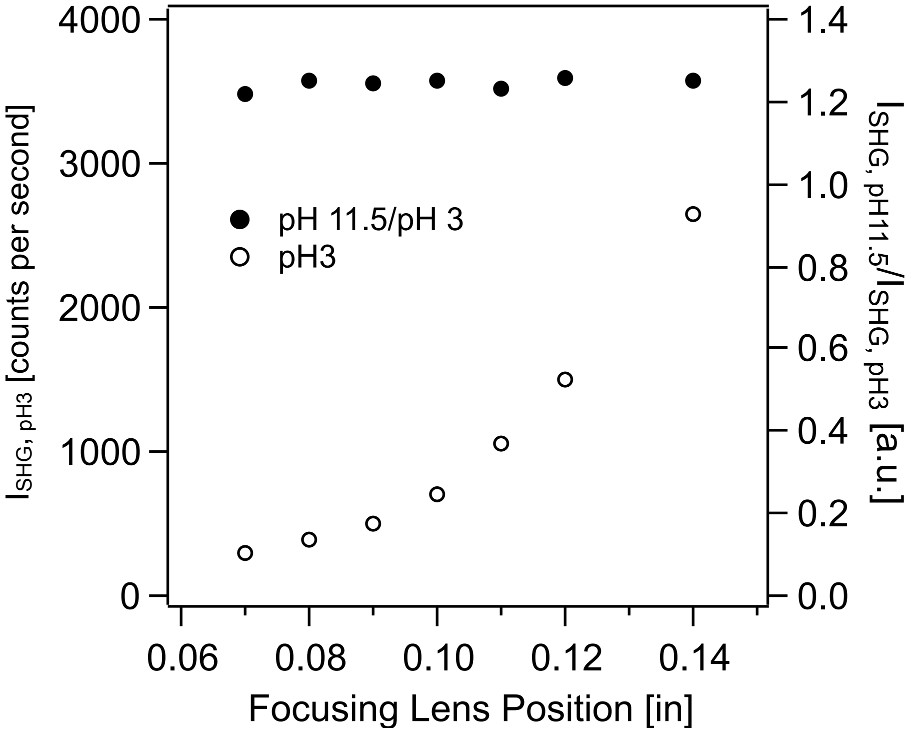

Supplementary Figure 4 | Signal intensity dependence upon focusing lens position. While the signal intensity at pH 3 (open circles, left axis), which is predominately from the bulk $\chi^{(2)}$ term of the α-quartz, is dependent upon the focusing lens position, the normalized jump intensity given by the signal at pH 11.5 divided by the signal at pH 3 (filled circles, right axis) is constant across a range of focusing lens positions that produce pH 3 signal intensities from ~300 to ~2600 counts per second. This allows attribution of changes in these normalized jump intensities across different rotational angles to the angles themselves as opposed to small changes in focusing lens position caused by physical rotation of an imperfectly aligned sample stage.

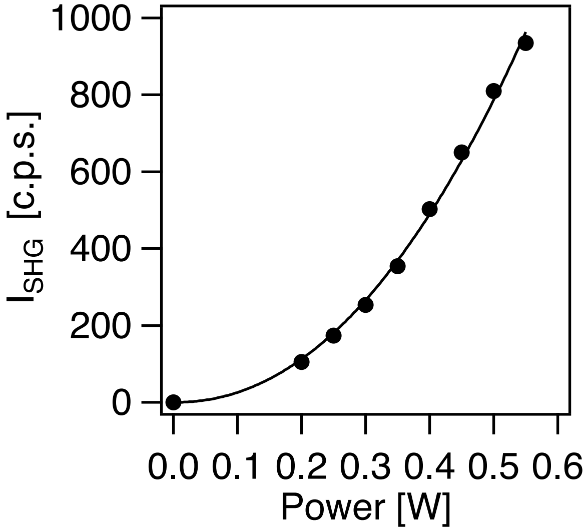

Supplementary Figure 5 | The power dependence of I_{SHG} for the α-quartz/water interface at pH 3; exponent in power fit = 2.1(1) as expected for a second order process such as SHG.

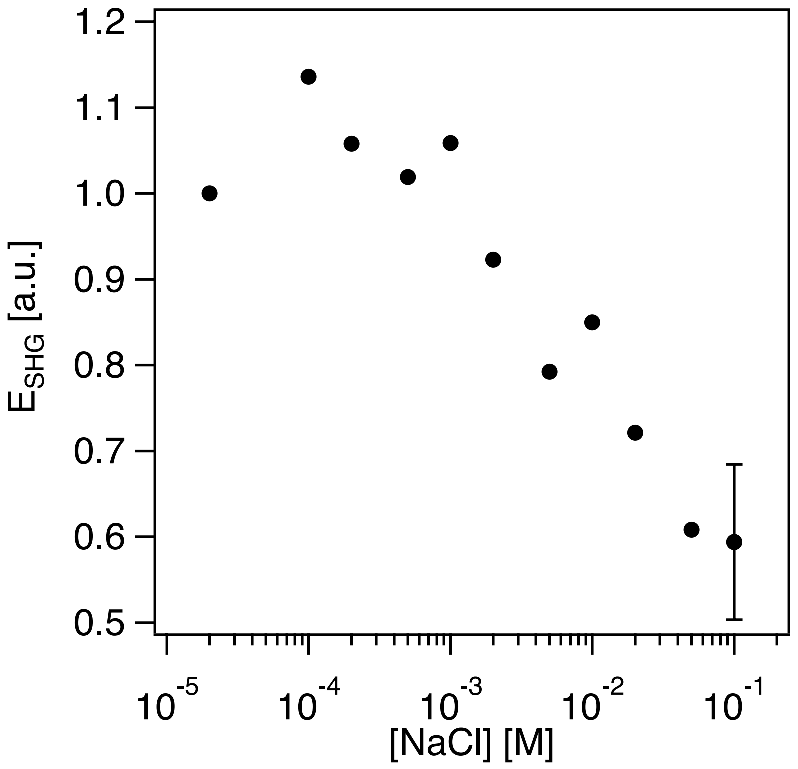

Supplementary Figure 6 | SHG intensity from the fused silica/water interface maintained at pH 7 during conditions of dynamically changing bulk solution ionic strength varying between 10^{-5} and 10^{-1} M NaCl. Interfacial potential and charge screening properties of the fused silica/water interface are expected to be comparable to those of the α-quartz/water interface, though the SHG signal lacks the bulk $\chi^{(2)}$ of the α-quartz due to the centrosymmetric nature of fused silica. This allows direct comparison to Figure 4 of the main text. Error bars represent one standard deviation with three measurements.

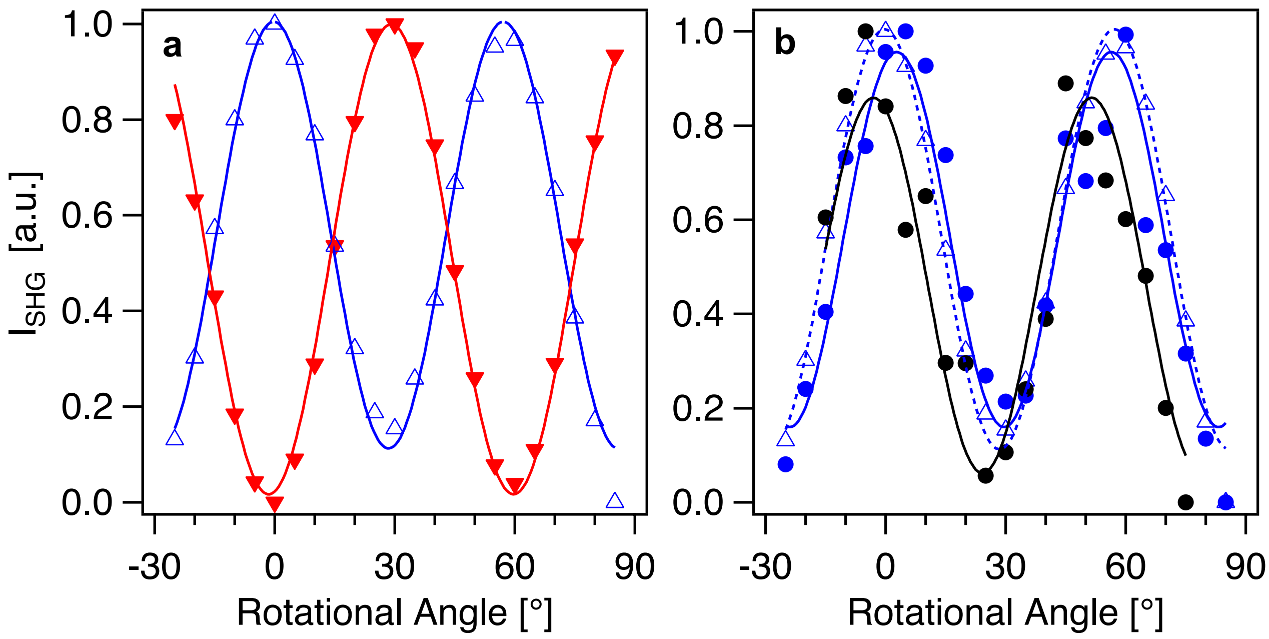

Supplementary Figure 7 | (a) Rotational angle dependence of I_{SHG} for PP (blue, open triangles) and PS (red, upside down closed triangles) of bulk α-quartz measured in air using the reflection geometry described in the Supplementary Methods section. (**b**) Rotational angle dependence of I_{SHG} in the PP polarization combination measured in air (blue, open triangles), with the hollow fused silica dome but no water present (blue, filled circles), and with the fused silica dome and pH 3 water present (black, filled circles), and using the reflection geometry described in the Supplementary Methods section. Imperfections in the spherical nature of the hand-blown dome introduces some error to the rotational dependence of the signal, but the periodic pattern is still present when going from the quartz/air (A) to the quartz/water (B) interface.

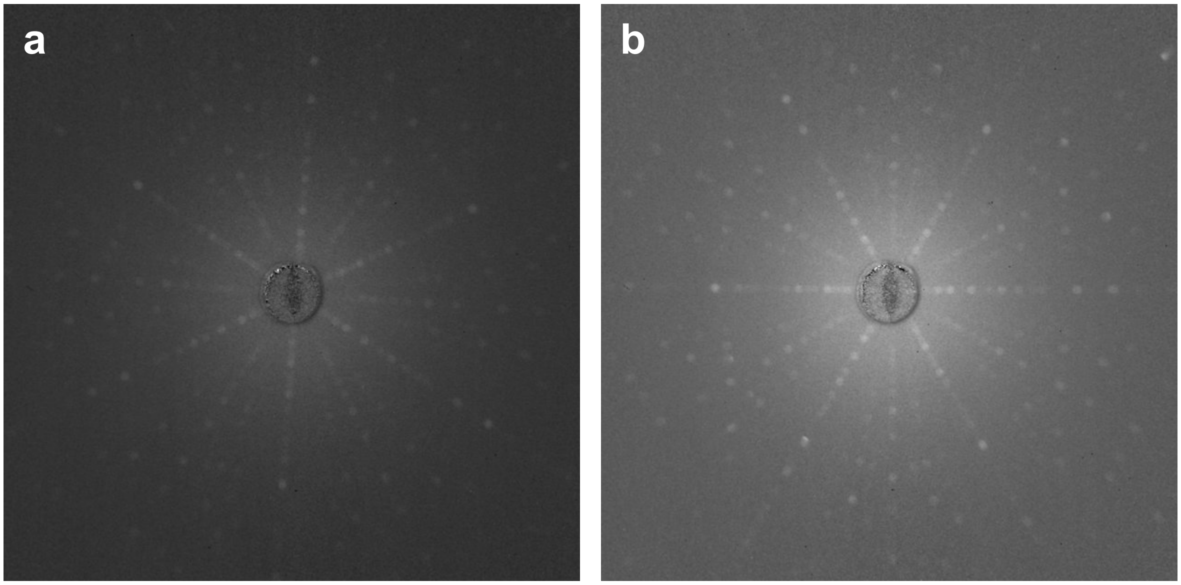

Supplementary Figure 8 | (a) Laue diffraction pattern of α-quartz samples of previously known absolute orientation (specimen from Knight Optical). (**b**) Laue diffraction pattern of α-quartz sample (specimen from Meller Optics) of unknown absolute orientation, used to identify the absolute orientation of the unknown orientation α-quartz crystal through direct comparison.

Supplementary Table 1 | Typical signal intensities measured using the internal and external reflection geometries schematically depicted in Supplementary Figure 1B. The internal reflection geometry gives higher signal intensities from both the bulk α-quartz and the fused silica surface at pH 11.5, as expected from the Fresnel factors.[4] However, if it is assumed that the fused silica and α-quartz surfaces have roughly comparable signal intensities at pH 11.5, it can be seen that the ratio of expected surface signal intensity to bulk signal intensity for α-quartz is more favorable in the external reflection geometry, motivating its experimental use in this study.

	**Typical I_{SHG} [c.p.s.]**	
	Internal Reflection	External Reflection
α-Quartz	1,500,000	1,000
Fused Silica	200	50
**Ratio**	**7,500**	**20**

Supplementary Note 1

A brief derivation of the origin of the $i\chi_2^{(3)}$ term, see also Refs. 32 and 33 in the Main Text.

As the electric field $E_{dc}(\mathrm{z}) = -d\Phi(\mathrm{z})/\mathrm{dz}$ is z (depth)-dependent and there is the phase matching factor that is also z dependent, one has:

$$\begin{aligned}
\chi_{dc}^{(2)} &= \int_0^\infty \chi^{(3)} E_{dc}(z) e^{-i\Delta k_z z} dz \\
&= \int_0^\infty -\chi^{(3)} \frac{d\Phi(\mathrm{z})}{dz} e^{-i\Delta k_z z} dz \\
&= -\chi^{(3)}\Phi(\mathrm{z})\mathrm{e}^{-i\Delta k_z z} \Big|_0^\infty + \chi^{(3)} \int_0^\infty \Phi(\mathrm{z})(\text{-}i\Delta k_z) e^{-i\Delta k_z z} dz \\
&= \chi^{(3)}\Phi(0) - i\Delta k_z \chi^{(3)} \int_0^\infty \Phi(\mathrm{z}) e^{-i\Delta k_z z} dz
\end{aligned} \tag{1}$$

Here, $1/\Delta k_z$ is the coherence length of the SHG or SFG process, $\Phi(\infty) = 0$, and the following integration relationship was used:

$$\int \frac{df(\mathrm{z})}{dz} g(\mathrm{z})\,\mathrm{dz} = f(z)g(z) - \int f(z)\frac{dg(\mathrm{z})}{dz} dz \tag{2}$$

A good approximation is that $\Phi(z) = \Phi(0)\mathrm{e}^{-kz}$, where $1/k$ is the Debye screening length factor. Then,

$$\begin{aligned}
\chi_{dc}^{(2)} &= \chi^{(3)}\Phi(0) - i\Delta k_z \chi^{(3)} \int_0^\infty \Phi(0) e^{-kz} e^{-i\Delta k_z z} dz \\
&= \chi^{(3)}\Phi(0) + \frac{-i\Delta k_z}{k + i\Delta k_z}\chi^{(3)}\Phi(0) \\
&= \frac{k}{k + i\Delta k_z}\chi^{(3)}\Phi(0)
\end{aligned}$$

Therefore, in the total effective surface susceptibility,

$$\chi_{eff}^{(2)} = \chi^{(2)} + \chi_{dc}^{(2)} = \chi^{(2)} + (\chi_1^{(3)} - i\chi_2^{(3)})\Phi(0) \tag{3}$$

one has

$$\chi_1^{(3)} = \frac{k^2}{k^2 + (\Delta k_z)^2} \chi^{(3)} \quad (4.1)$$

$$\chi_2^{(3)} = \frac{k \Delta k_z}{k^2 + (\Delta k_z)^2} \chi^{(3)} \quad (4.2)$$

Therefore, because the surface field is real and the phase matching factor is complex, the total $\chi_{dc}^{(2)} = (\chi_1^{(3)} - i\chi_2^{(3)})\Phi(0)$ contribution is complex.

When $k << \Delta k_z$, i.e. the Debye length is long (low electrolyte concentration), one finds

$$\chi_1^{(3)} \sim 0 \text{ and } \chi_2^{(3)} \sim \frac{k}{\Delta k_z} \chi^{(3)} \quad (5.1)$$

and the *dc* contribution is essentially imaginary.

When $k >> \Delta k_z$, i.e. the Debye length is very small (high electrolyte concentration), one finds

$$\chi_1^{(3)} \sim \chi^{(3)} \text{ and } \chi_2^{(3)} \sim \frac{\Delta k_z}{k} \chi^{(3)} \sim 0 \quad (5.2)$$

and the real term dominates.

When $k \sim \Delta k_z$, i.e. the Debye length and phase matching coherent length are comparable, the real and imaginary terms for the $\chi^{(3)}$ are comparable.

The derivation above assumes that the surface potential is of the form $\Phi(z) = \Phi(0)\,\mathrm{e}^{-kz}$. The actual surface potential may be different from this form, but essentially it decays when moving away from the surface. In addition, the surface potential can not only induce bulk $\chi^{(3)}$ responses from the water side, but also from the fused silica or the α-quartz side.[6] These issues warrant further investigation in the future.

Nevertheless, the following relationship, as established herein, should generally hold:

$$\chi_{eff}^{(2)} = \chi^{(2)} + \chi_{dc}^{(2)} = \chi^{(2)} + (\chi_1^{(3)} - i\chi_2^{(3)})\Phi(0) \quad (6)$$

Supplementary References

1 Achtyl, J. L., Unocic, R. R., Xu, L., Cai, Y., Raju, M., Zhang, W., ... & Geiger, F. M. Aqueous proton transfer across single-layer graphene. *Nature Communications* **6** (2015).

2 Geiger, F. M. Second Harmonic Generation, Sum Frequency Generation, and $\chi(3)$: Dissecting Environmental Interfaces with a Nonlinear Optical Swiss Army Knife. *Annual Review of Physical Chemistry* **60**, 61-83 (2009).

3 Ong, S. W., Zhao, X. L. & Eisenthal, K. B. Polarization of water molecules at a charged interface: second harmonic studies of the silica/water interface. *Chemical Physics Letters* **191**, 327-335 (1992).

4 Li, B., Li, X., Ma, Y.-H., Han, X., Wu, F.-G., Guo, Z., ... & Lu, X. Sum Frequency Generation of Interfacial Lipid Monolayers Shows Polarization Dependence on Experimental Geometries. *Langmuir* **32**, 7086-7095 (2016).

5 Gomez, S. A. S. & Geiger, F. M. Precipitates of Al(III), Sc(III), and La(III) at the Muscovite-Water Interface. *J. Phys. Chem. A* **118**, 10974-10981 (2014).

6 Bethea, C. G. Electric field induced second harmonic generation in glass. *Appl. Optics* **14**, 2435-2437 (1975).

7 Fu, L., Chen, S.-L. & Wang, H.-F. Validation of Spectra and Phase in Sub-1 cm–1 Resolution Sum-Frequency Generation Vibrational Spectroscopy through Internal Heterodyne Phase-Resolved Measurement. *J. Phys. Chem. B* **120**, 1579-1589 (2015).

8 Nowakowski, P. J., Woods, D. A., Bain, C. D. & Verlet, J. R. R. Time-resolved phase-sensitive second harmonic generation spectroscopy. *The Journal of Chemical Physics* **142**, 084201 (2015).
